# Post-activation Performance Enhancement Following Maximal Effort, Multi-Joint Isokinetic Eccentric Muscle Actions

**DOI:** 10.5114/jhk/200324

**Published:** 2025-05-12

**Authors:** Tom A. Dickey, Brennan J. Thompson, Cody M. Fisher, Tycen W. Flygare, Dale R. Wagner

**Affiliations:** 1 Kinesiology and Health Science Department, Utah State University, Logan, UT, USA.; 2 Movement Research Clinic, Sorenson Legacy Foundation Center for Clinical Excellence, Utah State University, Logan, UT, USA.

**Keywords:** vertical jump, countermovement jump, eccentric overload, Eccentron

## Abstract

Post-activation performance enhancement (PAPE) is a phenomenon that can enhance muscle performance following maximal or near-maximal muscle actions. While the effects of concentric and isometric conditioning actions on PAPE have been studied, less is known about the influence of eccentric muscle actions. This study investigated the effects of a multi-joint eccentric overload (EOL) protocol on PAPE expressed through countermovement jump (CMJ) height and isokinetic peak force (PF) outcome measures. Twenty-eight recreationally trained participants (18–30 years) completed three visits in a randomized, counterbalanced design. Following familiarization, participants performed either an EOL protocol involving two sets of six maximal isokinetic eccentric actions or a control condition (CON) involving cycling. The CMJ and PF were assessed at baseline and 15 s, 5 min, and 10 min post-exercise. Results showed no significant condition × time interaction or main effect of condition for either CMJ or PF (p > 0.05). However, a significant main effect of time (collapsed across condition) was observed for CMJ (p = 0.019), with post hoc analyses revealing a significantly higher CMJ at 5 min post-exercise compared to 15 s post-exercise (p = 0.037). These findings suggest that this multi-joint eccentric protocol did not effectively elicit PAPE, and therefore may not be optimal for inducing acute performance enhancement. Future research should further elucidate the optimal eccentric loading parameters and muscle action types for inducing PAPE.

## Introduction

Post-activation performance enhancement (PAPE) is a phenomenon that can enhance muscle performance following a maximal or near-maximal muscle action (Tillin and Bishop, 2009). When utilized appropriately, PAPE can enhance performance in explosive sports activities, such as sprinting or high jumping for example, in either competition or training (Docherty and Hodgson, 2007). Physiologically, post-activation potentiation (PAP) may result from the combination of phosphorylation of myosin regulatory light chains and the increased recruitment of higher-order motor units (Hodgson et al., 2005). Phosphorylation of myosin regulatory light chains increases calcium sensitivity at the site of cross-bridge formation, potentially leading to a greater rate of force development (RFD) (Hodgson et al., 2005). Notably, the mechanisms and attributes differ between PAP and PAPE, such that the phosphorylation aspect of muscle force augmentation is characterized by PAP, whereas the motor unit aspect is characterized by PAPE. The recruitment of higher-order motor units, which increases due to heightened excitability at the neuromuscular junction following intense muscle contractions, results in a greater RFD and force production (Hodgson et al., 2005). This is attributed to synaptic modifications at the spinal level that enhance motor unit firing rates and synchronization during subsequent contractions (Hodgson et al., 2005). Each physiological mechanism is thought to be elicited through different pathways, serving as a key factor in shaping the characteristics of various experimental protocols, as discussed later.

Maximal or near-maximal muscle contractions may lead to fatigue in addition to PAPE. In the immediate aftermath of a couple of sets of maximal contractions, fatigue has been found to dominate, but it dissipates faster than PAPE, thereby allowing PAPE (if fatigue is not too great) to become dominant and enhance performance (Güllich and Schmidtbleicher, 1996). Current research efforts are focused on understanding the interplay between PAPE and fatigue. Multiple factors intricately interact to modulate the magnitude and duration of the PAPE versus fatigue response, with the primary aim of minimizing fatigue and maximizing PAPE (Tillin and Bishop, 2009).

The relationship between PAPE and fatigue is complex and influenced by factors such as contraction intensity, volume, rest periods, contraction type, and individual characteristics like strength and fiber type. While maximal-intensity contractions are optimal for inducing PAPE (Tillin and Bishop, 2009), the volume should be kept low, with evidence suggesting no more than three sets should be done to minimize fatigue and maximize the PAPE effect (Hamada et al., 2003). Rest periods between sets directly impact volume, and a meta-analysis by Seitz and Haff (2015) suggests that 5–7 min of rest produces the greatest PAPE response. Additionally, stronger individuals tend to exhibit greater PAPE effect sizes, potentially due to a higher distribution of type II fibers and greater resistance to fatigue (Seitz and Haff, 2015).

Both concentric and isometric muscle action types affect the PAPE versus fatigue dynamics in different ways. One of the differences has to do with the fatigue profile of the muscle action types, where isometric actions may cause relatively more central fatigue due to the lack of blood flow to clear metabolic waste products from the action(s), thereby inhibiting alpha motor neuron activation and reducing the neural drive (Babault et al., 2006). However, concentric actions may lead to relatively more peripheral fatigue, where the dynamic nature of the muscle action can more effectively clear the metabolites out of the muscle due to more limited restriction in the blood flow (Tillin and Bishop, 2009). Still, lactate accumulation has been proposed to alleviate peripheral fatigue, such that by clearing lactate, central fatigue is reduced, but peripheral fatigue likely accumulates faster (Karelis et al., 2004). Focusing on the PAPE response, dynamic muscle actions are thought to elicit a PAPE response due to increased muscle spindle firing, thereby decreasing transmission failure from Iα sensory neurons and thus increasing higher-order motor unit activation (Tillin and Bishop, 2009). In comparison, isometric actions activate more motor neurons and may increase the percentage of myosin regulatory light chain phosphorylation (Tillin and Bishop, 2009), which, as noted earlier, involves more directly the PAP aspect of force augmentation. Both concentric and isometric muscle action types have had many studies showing a PAPE response and increases in performance in different types of activities (Bauer et al., 2018; Esformes et al., 2010; Esformes and Bampuras, 2013; Gahreman et al., 2020; Spieszny et al., 2022).

In comparison, fewer studies have looked at the effects of eccentric muscle actions when it comes to PAPE. One limited area where researchers have investigated eccentric actions is via the application of eccentric overload through flywheel devices, largely due to a lack of studies focusing on traditional fixed-resistance eccentric protocols in the context of PAPE. Flywheel training utilizes inertia, allowing resistance to vary based on the user's force output, which differs from the constant resistance found in traditional weightlifting exercises. While this difference in the mechanism may result in unique physiological responses, both modalities involve eccentric actions.

Beato and colleagues (2021b) found a significant PAPE response after a two-set flywheel eccentric overload (EOL) bout in the countermovement jump and lower body isokinetic strength, 3 to 9 min after the exercise bout. Additional work from Beato et al. (2021a) suggested that both medium and high-inertia flywheel squats produced similar PAPE results, indicating that multiple intensity levels could produce PAPE responses via flywheel exercise. In fact, even a single set of high-intensity flywheel EOL has produced a PAPE response (Maroto-Izquierdo et al., 2020). Although flywheel training is a unique modality, it offers valuable insight into the potential of eccentric overloading for inducing PAPE, highlighting the need for further research into traditional eccentric methods.

While several studies have examined the effects of flywheel eccentric overload actions on PAPE, there is limited research on the effects of pure eccentric actions without subsequent concentric action. Thus, it remains unknown whether the concentric or eccentric phase of the prior studies' experimental exercise protocols was more responsible for PAPE, or whether the combination of the two muscle action types is necessary to induce a PAPE response. Given that eccentric actions exhibit an increased capacity for higher force output than either concentric or isometric actions, it is possible that the eccentric-based enhanced force factor may be a primary driver of a heightened PAPE response. Moreover, the combination of the dynamic attribute with the eccentric mode along with the high loads may allow for an optimal scenario which allows for sufficient metabolite removal (like concentric) and higher force output (like isometric) to occur simultaneously. However, to address this question, the eccentric phase would need to be isolated, preferably in a multi-joint exercise model to examine the effects that an eccentric-only protocol may induce on a functional-based PAPE response. Therefore, this study aimed to investigate the effects of a multi-joint eccentric-overload bout on PAPE expressed through the vertical jump and eccentric (isokinetic) strength responses to gain a deeper understanding of the underlying influences on PAPE.

## Methods

### Experimental Design

This study utilized a randomized, counterbalanced repeated-measures design to investigate the effects of an experimental (ECC) protocol involving multi-joint isokinetic eccentric actions using a motor-driven eccentric isokinetic dynamometer (Eccentron, BTE Technologies Inc, Hanover, MD., USA) compared to a control (CON) condition involving a cycle ergometer on countermovement jumps (CMJs) and isokinetic strength (peak force; PF) as the primary outcomes. Participants arrived at the laboratory for three visits with 3–7 days in between visits, for a total of 1.5 h. On the first visit, participants reviewed and signed the informed consent document and were familiarized with the eccentric protocol on the Eccentron machine. Next, participants were randomly assigned to either the CON or ECC condition, and after 3–7 days, their condition assignment was counterbalanced for visit three.

### Participants

Twenty-eight college-aged men and women volunteered to participate in this investigation (mean ± SD: age = 20.6 ± 1.6 years; body height = 174.6 ± 6.5 cm; body mass = 77.1 ± 12.1 kg; 16 male, 12 female). Participants were required to be between the ages of 18 and 30 years and recreationally strength trained (the mean duration of strength training was 2.8 years for 2.3 hours per week), and must not have ever done eccentric-only strength training of the lower limbs. Recruitment consisted of flyers posted throughout a university campus and in-class announcements. Eligibility criteria required that participants be currently strength training the lower body, meaning they must have resistance trained their legs at least once a week, but no more than twice per week for the last three months at minimum and they must have had less than five hours of aerobic exercise per week. Participants were screened for underlying health conditions that could put them at risk during exercise, and they must not have had any lower leg injuries nor surgery within the last year. Sample size needs were estimated using G*Power (version 3.0.10; Heinrich Heine Universität Düsseldorf, Germany), where it was determined that to achieve a power level of 0.80, using an effect size of 0.25 (Beato et al., 2021b), and an alpha level of 0.05, a sample size of n = 22 would be required. To account for attrition, the study recruited 28 participants. The study was approved by the Institutional Review Board of Utah State University (protocol code: 12042; approval date: 02 September 2021), and all participants read and signed an informed consent document prior to any participation in the study.

### Familiarization Session

Participants underwent a screening process involving the eligibility criteria as described above and their body height and mass were measured using a wall stadiometer (Seca 216, Seca, Chino, CA) and a calibrated weight scale (Tanita WB-100A, Tanita Corp., Arlington Heights, IL) with shoes off for both measurements.

The warm-up session began with 5 min of stationary biking at 50 watts at 60 revolutions per min, followed by a series of 10 body weight squats and 3 CMJs performed at a self-selected 70% effort, followed by 2 unrecorded maximal CMJs (Beato et al., 2021b; Spencer et al., 2023). Participants were instructed to perform the CMJs with hands on their hips to prevent the influence of the arm swing on vertical jump performance and not to flex their knees before landing (Beato et al., 2021b). Participants were then seated on the Eccentron machine where the knee angle was measured with a goniometer at the most extended knee position (i.e., pedal forward) according to manufacturer guidelines. For the Eccentron, the excursion of the foot pedal is an absolute set distance per the machine’s design; however, on average, the knee angle range of motion was set for the point of most extension to ~35 degrees and following full pedal excursion (i.e., pedal back toward the participant), the end flexion point for the knee joint was ~97 degrees. This corresponded to a hip range of motion between ~87 and ~106 degrees, for the pedal movement from front to back, respectively (note 0 degrees = full extension). Participants then performed 2 submaximal effort sets of 6 reps per leg at a self-selected 30–50% effort, followed by 70% and ended with a maximum effort set, with 2-min rest intervals between sets. Isokinetic speed was set to 23 reps per min (Spencer et al., 2023).

### Sessions 2 and 3

The warm-up followed the same procedures as described in the familiarization session, minus the initial 30–50% set on the Eccentron. Two minutes following the warm-up, participants performed baseline testing consisting of 2 maximal CMJs using a Just Jump Mat (Just Jump Technologies, Huntsville, AL) and 2 maximal isokinetic repetitions on the Eccentron. Participants were randomized into either the ECC or CON conditions. For the ECC condition, participants performed a bout of two sets of eccentric maximal voluntary contractions (MVCs) on the Eccentron, which involved 2 sets of 6 MVCs per leg (12 in total), performed in an alternating, consecutive manner, with 2-min rest intervals between sets. For the CON condition, participants performed 5 min of stationary cycling at 60 revolutions per min at 1 watt/kg of body mass (Beato et al., 2021b). Participants were instructed to remain seated during the rest intervals. Upon completion of the eccentric protocol or stationary cycling, a timer was started and run for the duration of the follow up protocol.

Follow up testing was conducted at 15 s, 5, and 10 min post-exercise using the same procedures as the baseline tests. Next, participants were scheduled for the third and final session during which they performed the other condition.

### Data Acquisition

The CMJ height values from the Just Jump Mat were recorded manually. All raw force data (V) for maximal eccentric strength measures were collected using a data acquisition system (MP150WSW; Biopac Systems, Inc., Santa Barbara, CA, USA). Force data were sampled at 2000 Hz and processed offline with custom-written LabVIEW software (LabVIEW 2021, National Instruments, Austin, TX). The force signal was filtered using a fourth-order, zero-phase Butterworth filter at a low pass frequency of 50 Hz (Thompson et al., 2017). The highest value attained from the isokinetic eccentric MVCs was used for subsequent analyses.

### Statistical Analyses

Statistical analyses were conducted via SPSS software (version 25; IBM SPSS, Inc., Chicago, IL, USA) and utilized a two-way repeated measures analysis of variance (ANOVA) to test for differences between conditions (ECC × CON) as well as across four time points (baseline × 15 s × 5 min × 10 min). When appropriate, post-hoc analyses included Bonferroni-corrected pairwise comparisons. Descriptive statistics were reported as mean ± standard deviation and significance was determined using an alpha level of *p* < 0.05.

## Results

**Figure 1:**
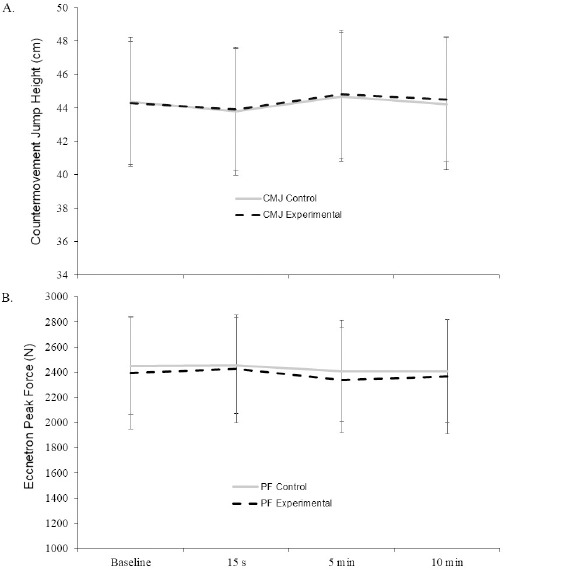
Countermovement jump (CMJ) and Eccentron peak force (PF) for the control and experimental conditions across all time points in the study. Data are mean ± SD.

All data are reported in Table 1 and illustrated in Figure 1. For the CMJ, there was no significant condition × time interaction (F (3, 81) = 0.417, *p* = 0.741) and no main effect for condition (F (1, 27) = 0.239, *p* = 0.629). However, a significant main effect of time was observed (F (3, 81) = 4.497, *p* = 0.019), with post hoc analyses revealing that the CMJ was significantly higher at time point 3 (5 min post-exercise) compared to time point 2 (15 s post-exercise) (*p* = 0.037). For PF, there was no significant condition × time interaction (F (3, 81) = 0.273, *p* = 0.844) and no main effect for condition (F (1, 27) = 1.215, *p* = 0.280) or time (F (3, 81) = 2.136, *p* = 0.102).

**Table 1:** Mean (SD) for countermovement jump height and peak force across conditions and time points.

Variable	Control	Eccentric
Baseline CMJ (cm)	44.35 (9.75)	44.29 (9.40)
15 s Post CMJ	43.79 (9.73)	43.89 (9.27)
5 min Post CMJ	44.65 (9.75)	44.81 (9.70)
10 min Post CMJ	44.22 (10.01)	44.53 (9.48)
Baseline PF (N)	2450.82 (390.48)	2391.06 (444.95)
15 s Post PF	2453.80 (383.34)	2424.82 (429.92)
5 min Post PF	2409.11 (402.68)	2336.66 (417.57)
10 min Post PF	2408.96 (407.79)	2365.88 (456.72)

Note: CMJ = countermovement jump; PF = Peak Force; N = Newtons

## Discussion

The primary findings of this investigation were that two sets of six maximal isokinetic eccentric muscle actions failed to elicit a PAPE response at any time period for the CMJ or PF, and there were no significant differences between the CON and ECC conditions.

While the current study did not find a significant PAPE effect following an eccentric-only conditioning protocol, other studies have investigated the effects of EOL exercises on PAPE using different eccentric structured protocols. Beato et al. (2021b) examined the effects of EOL flywheel half-squats on CMJ performance and lower-limb muscle strength. They found significant improvements in CMJ height, peak power, and peak force at various time points between 1 and 9 min post-exercise, as well as increased quadriceps and hamstrings isokinetic strength. However, their EOL protocol utilized a lower intensity (0.029 kg•m^−2^ flywheel inertia) compared to the maximal eccentric load used in the current study, which may partially explain the discrepancy in findings. Similarly, de Keijzer et al. (2020) found that 2 and 3 sets of EOL exercise significantly enhanced PAPE effects on both CMJ and long jump performance at 6 min post-exercise, while a single set did not. Although their study also used a lower intensity (0.029 kg•m^2^ flywheel inertia) and a different exercise modality (flywheel half-squats involving both eccentric and concentric phases) compared to the isolated eccentric actions in the current study, the combined findings underscore the potential role of EOL exercise volume in modulating PAPE responses.

Although the reason for the lack of a PAPE response in this investigation is unknown, one potential explanation is that the intensity of the eccentric conditioning actions may have been too high relative to the loads used in previous PAPE studies, which typically base their loads on concentric one repetition maximum (1RM). Eccentric actions can produce 20–60% greater force compared to concentric actions. The present study is rather unique in that it utilized a 1RM load based on maximal eccentric force. In contrast, many PAPE studies use loads ranging from 60 to 90% of concentric 1RM. For example, Bauer et al. (2019) used 60% and 90% of concentric 1RM in a back squat protocol and found PAPE effects on CMJ performance. Similarly, Seitz and Haff (2016) reported in a meta-analysis that PAPE effects were elicited using loads ranging from 60 to 84% of concentric 1RM. While there is not a direct conversion between concentric and eccentric modality 1RM loads, 100% of eccentric strength corresponds to approximately 120–160% of one's concentric 1RM (Franchi et al., 2017; Nuzzo et al., 2023). While the flywheel studies by Beato et al. (2021a and 2021b) did apply eccentric overloading, the inertial loads used were likely lower than the 100% eccentric 1RM used in the current study. Consequently, the high intensity eccentric muscle actions used in our protocol, when compared to the relatively lower intensities typically used in PAPE studies based on concentric 1RM, might have induced excessive fatigue, counteracting any potential potentiation effects.

In connection with the relatively heightened intensity, one may observe the resulting higher volume of the eccentric conditioning actions here, as perhaps compared to other PAPE investigations. Although our protocol utilized a similar number of sets and repetitions (2 sets of 6 repetitions) as previous studies that have demonstrated PAPE effects (de Keijzer et al., 2019), the relatively higher intensity (100% eccentric 1RM) from the eccentric actions would have resulted in greater overall load/volume. Tillin and Bishop (2009) suggest that too high volumes of conditioning contractions can lead to excessive fatigue, which may counteract the potential benefits of PAPE. Indeed, achieving the optimal balance of the load/volume for eliciting PAPE is challenging, as fatigue may overcome potentiation when the optimal load/volume is exceeded. For instance, de Keijzer et al. (2019) reported that 2 sets of flywheel eccentric overload exercise were required to induce PAPE effects on CMJ and long jump performance, while a single set was not enough. However, it is important to note that the intensity used in their study (0.029 kg•m^−2^ flywheel inertia) was likely lower than the 100% eccentric 1RM used in our protocol. Consequently, the combination of higher relative intensity from the eccentric-based training intensities used, and multiple sets in our study might have resulted in an excessively high volume/load, leading to fatigue that may have overshadowed any potential PAPE effects.

The underlying mechanisms may also have been a key factor for the present findings resulting from the eccentric-only protocol. One of the proposed mechanisms of PAP is the phosphorylation of myosin regulatory light chains (RLCs), a process that requires calcium release from the sarcoplasmic reticulum (Sweeney et al., 1993). However, eccentric muscle actions have been shown to have unique calcium dynamics and have been associated with a lower energy cost and distinct neural activation strategies compared to concentric actions (Franchi et al., 2017). Consequently, the lower calcium transient during eccentric muscle actions could lead to reduced phosphorylation of myosin RLCs, thereby attenuating the PAP response. Therefore, it is possible that the unique calcium dynamics and excitation-contraction coupling responses that are specific to eccentric-only actions may explain the lack of PAPE effect observed in the present study. However, more research is needed to investigate these proposed mechanisms regarding eccentric-based protocols and PAPE.

Another potential explanation for the lack of PAPE effect in the present study is the possibility of reduced firing rates of high-threshold motor units following the maximal eccentric actions. Farup et al. (2016) demonstrated that the firing rates of later recruited, high-threshold motor units were significantly reduced following maximal eccentric exercise, while the firing rates of early- and mid-recruited motor units remained unchanged. Similarly, Balshaw et al. (2017) found a decline in the firing rates of high-threshold motor units following eccentric overload muscle actions, despite no changes in MVC force. These findings would corroborate this theory such that eccentric actions, particularly when performed at high eccentric intensities and overload as in the present study, may selectively impair the firing rates of high-threshold motor units. Given that high-threshold motor units are preferentially recruited during high-force muscle actions and are associated with fast-twitch muscle fibers, a selective reduction in their firing rates following eccentric exercise could potentially attenuate the PAPE response. The attenuation of high-threshold motor unit firing rates following eccentric actions (Balshaw et al., 2017; Farup et al., 2016) could potentially explain the lack of a PAPE effect in the current study, as the eccentric-only preconditioning protocol may have selectively impaired the firing rates of the motor units most responsible for explosive force production. However, further research is needed that investigates these motor unit-based mechanisms with high intensity eccentric-only protocols.

Another possible explanation for the lack of PAPE in the present study is that eccentric muscle actions are known to cause muscle damage, particularly at high intensities or volumes, which can lead to decrements in force production (Howatson and van Someren, 2008; Proske and Allen, 2005). In the present study, although the eccentric conditioning protocol involved a relatively low volume (2 sets of 6 repetitions), the high relative intensity (100% of eccentric 1RM) might have induced some level of muscle damage. This damage, even if minimal, could have contributed to the lack of the PAPE effect observed. Muscle damage can impair force transmission and excitation-contraction coupling (Proske and Morgan, 2001), potentially offsetting any potentiating effects of the conditioning actions. However, it is important to note that the extent of muscle damage and its impact on performance can vary depending on factors such as the exercise protocol, muscle group, and individual characteristics (Nosaka and Aoki, 2011). Given that our protocol involved a relatively low volume and that participants were resistance-trained individuals, the role of muscle damage in the observed outcomes would be expected to be limited, although it may have still been minimally present enough to have a small PAPE attenuating effect. Nevertheless, to examine this plausible mechanism, future studies could directly assess markers of muscle damage following similar eccentric conditioning protocols to better understand its potential influence on PAPE responses. Finally, it is possible that the range of motion as implemented in this study had an influence on these outcomes, as prior work has shown that the range of motion of the conditioning movement influences the PAPE response (Esformes and Bampuras, 2013).

While the present study focused on the PAPE response following isolated eccentric conditioning actions, it is worth noting that most previous studies investigating eccentric exercise have utilized protocols involving both eccentric and concentric actions, such as flywheels or the lowering and lifting phases of a squat or a bench press (Esformes et al., 2010). Therefore, it is possible that the absence of a subsequent concentric action following the eccentric conditioning actions in our protocol could have influenced the PAPE response. However, to our knowledge, only one other study to date has used a protocol completely lacking the concentric action (Stastny et al., 2024). Thus, this study provides an important contribution to the literature by examining the PAPE effects from an eccentric-only conditioning protocol, without any influence of the concentric muscle action.

Recent research by Stastny et al. (2024) provides intriguing insights into the role of eccentric conditioning actions in PAPE, offering a valuable comparison to our own findings. Their study investigated the effects of different eccentric velocities during front squat conditioning activities on subsequent hip and thigh muscle performance. While our study on eccentric-only muscle actions did not find significant improvements in CMJ height or PF compared to a control group, Stastny et al. (2024) observed some notable PAPE effects, particularly with their 2-s eccentric-only protocol (FSqE2s). Interestingly, they also found no significant increase in jump height, aligning with our results. However, they did report significant improvements in PF and the RFD during the CMJ, as well as enhanced performance in specific muscle groups, especially hip extensors and knee flexors. This discrepancy in findings might be attributed to differences in the conditioning protocol, such as the use of front squats instead of isolated muscle actions, or the specific performance measures examined. Their study also compared the eccentric-only protocol with a fast eccentric-concentric protocol, finding the eccentric-only approach more effective in eliciting PAPE for certain outcomes. These results suggest that while eccentric-only protocols may not consistently improve global measures such as jump height, they may enhance specific force production characteristics and targeted muscle group performance, something our study did not measure. It should also be noted that although the present study did not show a PAPE effect, it also did not induce any detrimental effect either. Thus, using the specific eccentric exercise parameters in this study, no excess fatigue was induced leading to reduced performance. To better understand the physiological and functional impact of this specific modality of exercise on PAPE, future research should further investigate the nuances of eccentric-only protocols, including exercise selection, loading variables, and their effects on various performance measures, to better understand the conditions under which PAPE can be optimally achieved.

The only statistically significant finding in this study was a main effect of time (collapsed across groups) on CMJ performance, with post hoc analyses revealing that CMJ height was significantly higher at 5 min post-exercise compared to 15 s post-exercise. Various factors could potentially influence this time-dependent change in CMJ performance, such as the recovery of the stretch-shortening cycle, the dissipation of muscle fatigue, or changes in neuromuscular activation patterns. Recent studies by Nakata and Mishima (2024) and Pojskic et al. (2024) explored the effects of different conditioning activities on PAPE. Nakata and Mishima (2024) examined jump exercises and found that, while PAP was present up to 2 min after the activity, it did not significantly improve the RFD, underscoring the complex relationship between PAP and performance outcomes. Similarly, Pojskic et al. (2024) investigated the effects of whole-body vibration on CMJ performance and found no significant differences between loaded and unloaded interventions. However, they highlighted the importance of a 4-min rest interval to optimize performance, emphasizing the critical role of rest duration in PAPE responses. Together, these findings illustrate the variability in PAPE responses and underscore the importance of the exercise type, conditioning activity, and recovery duration in performance outcomes. It is important to note that this time effect of our study was observed regardless of the conditioning protocol (ECC or CON), suggesting that the recovery process itself, rather than the specific intervention, may have been the primary driver of the observed improvement in CMJ height. However, further research is needed to elucidate the specific mechanisms underlying this time-dependent effect on CMJ performance in the context of EOL protocols. It should also be noted that a recent systematic review by Younes-Egana et al. (2023) highlights the effectiveness of eccentric overload training in enhancing CMJ and sprint performance, providing additional evidence for the potential benefits of eccentric training on athletic performance outcomes, that may be prevalent even without PAPE responses.

The present investigation has some limitations that are noteworthy. First, the study involved a relatively small sample of recreationally trained individuals, which may limit the generalizability of the findings to other populations, such as highly trained athletes or sedentary populations. Second, the study only examined the acute PAPE response to a single eccentric conditioning protocol; future research should investigate the effects of different eccentric loading parameters and training duration on PAPE (for example, a future study could look at the effects of a single set of six maximal eccentric muscle actions rather than two sets). Third, the study did not directly assess markers of muscle damage or fatigue, which could have provided additional insight into the mechanisms underlying the observed lack of PAPE effect. Fourth, none of the participants had previous experience with eccentric training, which could significantly impact the results. Finally, it should be noted that the eccentric protocol conducted in this study was performed in a unilateral (alternating legs) manner. Therefore, the results of this study are specific to a unilateral exercise design, and future work is needed to evaluate how unilateral training may differ from bilateral (simultaneous) training due to specificity factors such as fatigue, neuromuscular activation differences, workloads etc. Despite these limitations, the present study provides valuable insights into the PAPE response to maximal isokinetic eccentric conditioning actions and highlights potential areas for future research.

## Conclusions

In summary, the present study found that two sets of six maximal isokinetic eccentric conditioning actions failed to elicit a PAPE response in recreationally trained individuals. Several potential explanations for this lack of PAPE effect include the high relative intensity and, consequently, greater load/volume of the eccentric conditioning actions as compared to concentric-focused studies, the unique calcium dynamics and excitation-contraction coupling responses that are inherent to eccentric muscle actions, potentially reduced firing rates of high-threshold motor units following maximal eccentric exercise, and the potential role of muscle damage. In practice, it does not appear that performing multi-joint eccentric conditioning repetitions has any benefit for improving functional (vertical jump) or strength-based measures in the minutes that follow the protocol.
